# Annotations

**Published:** 1897-10-23

**Authors:** 


### Annotations.

### The New Member of the General Medical Council.

The result of the polling for a direct representative on the General Medical Council was as follows: Mr. Victor Horsley, 6,946; Sir Walter Foster, 6,112; Dr. Rigby, 197; Dr. Diver, 81. Mr. Victor Horsley is to be congratulated on his success, and the medical profession is to be doubly congratulated on having had the good sense to return the best candidate in the field, quite irrespective of his place of residence, and his department of practice. We shall watch with no small interest the temper and the manner of his reception by his colleagues. That he will have certain difficulties to contend with goes almost without saying. It is generally recognised that Mr. Horsley enters Council with the intention not only of promoting medical reform in general, but of reforming the Council itself and all its doings, and promoters of such reforms are not always loved. Moreover, Mr. Horsley may well be termed a "strong" representative. When he discovers what he considers an abuse, he does not hesitate to say so; and perhaps he has at times expressed his opinions in terms which might have been modified with advantage—at any rate, so far as his personal acceptability is concerned, so that it is quite easy to believe that his seat at the Council may not be altogether stuffed with rose leaves. So far as his own personality is concerned, we can have no doubt that Mr. Horsley will show that *savoir faire* and that urbanity which will make him an acceptable colleague, and thus belie the gloomy anticipations of some who look upon him as a firebrand; but we feel quite sure at the same time that he will not smooth his seat at the expense of his principles. His programme is perfectly plain; his mandate (if one may use such a term in regard to a man who has been accepted on account of his declared opinions) is perfectly definite and one of the main reasons of his being elected is that his constituents have regarded him as the best man to "bell the cat." It is, in fact, because his supporters dislike the Medical Acts and distrust the Council that Mr. Horsley has been returned; and it would be idle to expect either that he will be able to confine himself to smooth sayings, or that he will be received with open arms. These, however, are but details; he has certain things to do, and we have no doubt that he will do them with all his heart. He is the last man in the world to be tamed by office, while from his position as a surgeon, as a teacher, as a man of science, and finally as a direct representative of the medical profession, it will be impossible for his colleagues to ignore him.

### Night Caps.

In days of old the upper chambers of many country houses were more or less open to the roof, and even in those nearer to centres of society bedrooms were often vast and gusty apartments, in which the windows rattled and the winds of heaven played mad pranks throughout the livelong night. One need not wonder, then, that in those days the four-post bed was a popular institution, modestly ensconced within the walls of which the sleeper slept in peace. But even that was not enough to make our great grandparents comfortable at night. Perhaps the monstrous wig of the Georgean period may have had something to do with it—perhaps the change from wig to nakedness was more than even their heads could bear; in any case, the night cap was quite an essential feature of the night costume. Of course we have changed all that. Our chimneys are furnished with registers, our windows are made to fit, our doors are padded up with mats and covered over with portières, so that modern housekeepers now point with pride to their curtainless brass bedsteads, and boast of their great sanitary triumph in getting rid of "those nasty hangings," and modern men and women are able to sleep without their night caps! What we would point out, however, is that the abolition of the night cap, advantageous as it may be from an æsthetic point of view, is dearly bought at the price of lack of ventilation of the bedroom. In the daytime we enjoy the coldest air. No one in reasonable health objects to cold so long as he is well wrapped up. The real difficulty is the carrying of the weight of clothes. But at night this need be no difficulty at all. There is no limit to the wraps we can pile on; only, if we would get the full benefit of fresh air at night, the head should be clothed as well as the body. A good fire to go to bed with, and open windows afterwards, are immense aids to health, but to gain their full utility without discomfort we want the night cap.

### A Nasty, Wasteful Habit.

Attention has been called to the dangers attending the sale of "chewing gum" by an inquest which has been recently held at Lincoln on a child aged between seven and eight years old, who died after eating this substance, which it not unnaturally imagined was a sweetmeat. We would point out, however, that, besides such risks as this, the habit of masticating this filthy compound of flavoured indiarubber is undoubtedly a cause of much dyspepsia. The constant titillation of the salivary organs kept up by chewing this stuff not only causes a steady drain of saliva, which is most wasteful, but, what is more serious still, in consequence of the frequently-repeated stimulation to which these organs are thus exposed, they fail to respond to the normal excitation which ought to rouse them to action when food is taken. A constant dribble of salivary secretion is substituted for the healthy flow which should occur only at meal times. The glands fail to respond to any stimulant less potent than the peppermint, aniseed, or other constituents found in chewing gum; and the more insipid foods, such as bread and other starchy compounds, pass into the stomach unchanged. This is disturbing to digestion at its very commencement, and it is extremely probable that the indigestion for starchy substances, which is so commonly met with at the present day, is largely due to the waste of saliva caused by smoking and by the constant chewing of various substances, which we see going on all around. The chewing of gum is thus not only a nasty habit, but is provocative of ill-health. Unfortunately, when "chewing gum" is sold in the form of a sweetmeat it may cause still more serious consequences, being apt to be swallowed by children, who, like their first parents, when they see that it is apparently good for food and pleasant to the eyes, are undeterred by the superscription "not to be eaten."

